# One-Pot Synthesis of **β**-Acetamido Ketones Using Boric Acid at Room Temperature

**DOI:** 10.1100/2012/925617

**Published:** 2012-04-29

**Authors:** Zahed Karimi-Jaberi, Korosh Mohammadi

**Affiliations:** Department of Chemistry, Firoozabad Branch, Islamic Azad University, P.O. Box 74715-117, Fars, Firoozabad, Iran

## Abstract

**β**-acetamido ketones were synthesized in excellent yields through one-pot condensation reaction of aldehydes, acetophenones, acetyl chloride, and acetonitrile in the presence of boric acid as a solid heterogeneous catalyst at room temperature. It is the first successful report of boric acid that has been used as solid acid catalyst for the preparation of **β**-acetamido ketones. The remarkable advantages offered by this method are green catalyst, mild reaction conditions, simple procedure, short reaction times, and good-to-excellent yields of products.

## 1. Introduction

During the last few years, multicomponent reactions (MCRs) have proved to be remarkably successful in generating molecular complexity in a single synthetic operation. These processes consist of two or more synthetic steps, which are performed without isolation of any intermediates, thus reducing time and saving both energy and raw materials. MCRs are powerful tools in the modern drug discovery process and allow fast, automated, and high throughput generation of organic compounds. Furthermore, a field of increasing interest is the synthesis of useful synthetic building blocks via MCRs chemistry. For this reason, the discovery of novel MCRs is of interest [1–3].

*β*-acetamido ketones are versatile intermediates in that their skeletons exist in a number of biologically or pharmacologically important compounds [4]. The best known route for the synthesis of these compounds is the Dakin-West reaction [5], which involves the condensation of an amino acid with acetic anhydride in the presence of a base via an intermediate azlactone to give the acetamido ketones [6]. Bhatia et al. proposed another procedure for the formation of these compounds through the condensation of an aryl aldehyde, acetophenone, and acetyl chloride in acetonitrile in the presence of CoCl_2_ [7] or montmorillonite K-10 clay [8]. Other catalysts such as heteropolyacids [9], HClO_4_-SiO_2_ [10], CeCl_3_ [11], ZnO [12], cyanuric chloride [13], Amberlyst-15 [14], and POCl_3_/Borax [15] have been used. Although these methods are valuable, most of them employ expensive catalysts, long reaction times, or harsh reaction conditions. Therefore, the introduction of new and efficient methods for this multicomponent reaction is still necessary. 

Following our systematic studies directed toward the development of practical, safe, and environmentally friendly procedures for several important organic transformations [16–19], herein we describe an efficient method for the synthesis of *β*-acetamido ketones through the condensation of an aryl aldehyde, an acetophenone, acetyl chloride, and acetonitrile in the presence of boric acid at room temperature.

## 2. Results and Discussion

Boric acid (H_3_BO_3_) is a useful and environmentally benign catalyst which has been successfully utilized in numerous reactions, for example, the aza-Michael addition [20], Biginelli reaction [21], transesterification of ethyl acetoacetate [22], Mannich reaction [23], and by our group in the synthesis of dibenzoxanthenes [16] and *α*-aminophosphonates [17]. It offers milder conditions relative to common mineral acids. Boric acid is a readily available and inexpensive reagent and can conveniently be handled and removed from the reaction mixture. Thus, the remarkable catalytic activities together with its operational simplicity make it the most suitable catalyst for the synthesis of *β*-acetamido ketones.

**Scheme 1:**
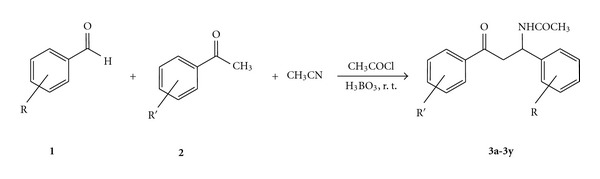


To optimize the reaction conditions, the reaction of benzaldehyde, acetophenone, acetyl chloride, and acetonitrile was used as a model reaction. The optimized reactant ratios were found to be 1.0 equiv. benzaldehyde, 1.0 equiv. acetophenone, 2 equiv. acetyl chloride, 4 mL acetonitrile, and 0.1 g of boric acid. The expected product was produced in 95% yield after 1.5 h at room temperature (Scheme 1). After completion of the reaction, the catalyst (boric acid) can easily be separated from the reaction mixture. To show the generality and scope of the boric acid-promoted *β*-acetamido ketones synthesis, the reaction was examined with various structurally diverse aldehydes and acetophenones. The results are summarized in Table 1. The structures of products were confirmed by comparison with their known physical and spectral (NMR and IR) data [6–13].

**Scheme 2:**
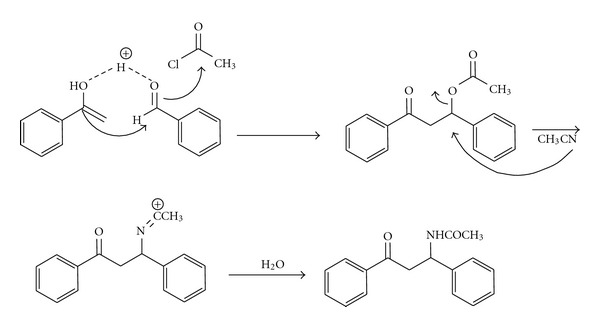


The suggested mechanism for this reaction is patterned in Scheme 2. We believe that boric acid is coordinated to the oxygen of the aldehyde and activate benzaldehyde for nucleophilic attack [11]. On the other hand, boric acid facilitates enolization of the acetophenone. The presence of acetyl chloride is necessary for the transformation since the reaction in its absence gave none of the desired product even after 2 h. It is important to note that the synthesis of *β*-acetamido ketones could not be achieved in the absence of catalyst (boric acid).

## 3. Conclusions

In conclusion, this paper describes a convenient and efficient process for the synthesis of *β*-acetamido ketones through the condensation of an aryl aldehyde, an acetophenone, acetyl chloride, and acetonitrile in the presence of boric acid as a solid heterogeneous catalyst at room temperature. This method offers some advantages in terms of simplicity of performance, low reaction times, solvent-free condition, low cost, and it follows along the line of green chemistry. The catalyst is readily available and inexpensive and can conveniently be handled and removed from the reaction mixture. We believe that this procedure is convenient, economic, and a user-friendly process for the synthesis of *β*-acetamido ketones of biological and medicinal importance.

## 4. Experimental

### 4.1. General Procedure for Preparation of *β*-acetamido Ketones

A mixture of aldehyde (2 mmol), acetophenone (2 mmol), acetyl chloride (2 mmol), acetonitrile (4 mL), and boric acid (0.1 g) was stirred at room temperature for the appropriate time indicated in Table 1. The progress of reactions was monitored by TLC (ethyl acetate/n-hexane = 1/4). After completion of the reaction, the reaction mixture was diluted with water and extracted with ethyl acetate, concentrated under vacuum, and the crude mixture was purified by recrystallization from ethyl acetate/n-hexane to give the pure product.

### 4.2. Spectral Data for Selected Products

#### 4.2.1. *β*-acetamido-*β*-(4-chlorophenyl)-4-chloropropiophenone 3f

(Table 1, entry 6) mp 141–143°C, IR (KBr, cm^−1^): 3264, 3056, 1670, 1635, 1584, 1292, 1088, 885, 825, ^1^HNMR (CDCl_3_): *δ* 2.08 (s, 3 H), 3.40 (dd, *J* = 7.3, and 10.9 Hz, 1 H), 3.82 (dd, *J* = 7.3, and 10.9 Hz, 1 H), 5.57 (m, 1 H), 7.32 (s, 1 H), 7.47 (d, *J* = 9.1 Hz, 4 H), 7.90 (d, *J* = 9.1 Hz, 4 H).

#### 4.2.2. *β*-acetamido-*β*-(2,3-dichlorophenyl)-4-chloropropiophenone 3t

(Table 1, entry 20) mp 200–202°C, IR(KBr, cm^−1^): 3291, 3077, 1690, 1651, 1589, 1547, 1401, 1370, 1298, 1225, 1197, 1091, 996, 816, 785, 742, 657. ^1^HNMR (CDCl_3_): *δ* 2.03 (s, 3 H), 3.42 (dd, *J* = 5, and 17.5 Hz, 1 H), 3.72 (dd, *J* = 5, and 17.5 Hz, 1 H), 5.82 (m, 1 H), 6.96 (d, br, *J* = 7.5 Hz, 1 H), 7.12–7.43 (m, 5 H), 7.82 (d, *J* = 7.5 Hz, 2 H). ^13^CNMR (CDCl_3_): *δ* 23.33, 41.01, 48.49, 126.46, 127.38, 129.10, 129.54, 133.56, 134.57, 140.34, 169.44, 197.45.

## Figures and Tables

**Table 1:** Synthesis of *β*-acetamido ketones using boric acid.

Entry	R	R′	Product	Time (h)	Yield (%)
1	H	H	**3a**	1.5	95
2	H	4-Cl	**3b**	1.5	90
3	H	4-NO_2_	**3c**	3.5	97
4	H	4-CH_3_	**3d**	2	85
5	4-Cl	H	**3e**	2	88
6	4-Cl	4-Cl	**3f**	2	88
7	4-Cl	4-NO_2_	**3g**	2	90
8	4-Cl	4-CH_3_	**3h**	1.5	88
9	4-CH_3_	H	**3i**	1.5	95
10	4-CH_3_	4-CH_3_	**3j**	2	92
11	4-CH_3_	4-NO_2_	**3k**	2	98
12	3-NO_2_	H	**3l**	3	90
13	3-NO_2_	4-Cl	**3m**	3.5	92
14	4-NO_2_	H	**3n**	3.5	91
15	4-NO_2_	4-Cl	**3o**	2	92
16	4-NO_2_	4-NO_2_	**3p**	3	87
17	4-OH	H	**3q**	0.5	80
18	4-CH_3_O	H	**3r**	0.5	86
19	2,3-Cl_2_	H	**3s**	2.5	87
20	2,3-Cl_2_	4-Cl	**3t**	2	92
21	2,4-Cl_2_	4-NO_2_	**3u**	3.5	85
22	2,6-Cl_2_	H	**3v**	3	80
23	2-Cl-6-F	H	**3w**	4.5	80
24	2-Cl-6-F	4-NO_2_	**3x**	4	88
25	4-Cl-3-NO_2_	H	**3y**	2	90
